# Actomyosin-Dependent Cortical Dynamics Contributes to the Prophase Force-Balance in the Early *Drosophila* Embryo

**DOI:** 10.1371/journal.pone.0018366

**Published:** 2011-03-31

**Authors:** Patrizia Sommi, Dhanya Cheerambathur, Ingrid Brust-Mascher, Alex Mogilner

**Affiliations:** 1 Human Physiology Section, Department of Physiology, University of Pavia, Pavia, Italy; 2 Department of Cellular and Molecular Medicine, Ludwig Institute for Cancer Research, University of California San Diego, La Jolla, California, United States of America; 3 Department of Molecular and Cellular Biology, University of California Davis, Davis, California, United States of America; 4 Department of Neurobiology, Physiology and Behavior, University of California Davis, Davis, California, United States of America; 5 Department of Mathematics, University of California Davis, Davis, California, United States of America; Institute of Science and Technology Austria, Austria

## Abstract

**Background:**

The assembly of the *Drosophila* embryo mitotic spindle during prophase depends upon a balance of outward forces generated by cortical dynein and inward forces generated by kinesin-14 and nuclear elasticity. Myosin II is known to contribute to the dynamics of the cell cortex but how this influences the prophase force-balance is unclear.

**Principal Findings:**

Here we investigated this question by injecting the myosin II inhibitor, Y27632, into early *Drosophila* embryos. We observed a significant increase in both the area of the dense cortical actin caps and in the spacing of the spindle poles. Tracking of microtubule plus ends marked by EB1-GFP and of actin at the cortex revealed that astral microtubules can interact with all regions of these expanded caps, presumably via their interaction with cortical dynein. In *Scrambled* mutants displaying abnormally small actin caps but normal prophase spindle length in late prophase, myosin II inhibition produced very short spindles.

**Conclusions:**

These results suggest that two complementary outward forces are exerted on the prophase spindle by the overlying cortex. Specifically, dynein localized on the mechanically firm actin caps and the actomyosin-driven contraction of the deformable soft patches of the actin cortex, cooperate to pull astral microtubules outward. Thus, myosin II controls the size and dynamic properties of the actin-based cortex to influence the spacing of the poles of the underlying spindle during prophase.

## Introduction

Microtubule (MTs) and actin-myosin arrays interact and cooperate in many mechanochemical modules of cell motility and cell division [1] but the functional implications of such interactions are not well understood. In particular, interactions of mitotic spindles with the F-actin cortex are crucial for spindle positioning and orientation [2]–[4] as well as the regulation of cytokinesis [5], yet whether the actin-myosin network affects internal processes of mitotic spindle assembly and maintenance, or only external phenomena involving the spindle's interactions with other regions of the cell such as the cortex, is still a controversial question [4]. Some evidence suggests that myosin II is needed only for cytokinesis: inhibition of myosin II in echinoderm blastomeres blocks cytokinesis but not mitosis [6]; similarly, RNAi depletion of myosin II in S2 cells blocks cytokinesis but metaphase and anaphase spindles are morphologically normal [7].

On the other hand, myosin II has recently been reported to exert force on the spindle poles during prophase, presumably via a drag on cortex-anchored astral microtubules subsequent to nuclear envelope breakdown (NEB) through myosin-powered cortical flow [2]. In locust spermatocytes, there is evidence that actin and myosin are involved in anaphase chromosome movement [8]. Curiously, it was recently reported that F-actin promotes spindle lengthening, perhaps by interactions with astral MTs, while Myosin-10 works antagonistically to shorten the spindle [9].

The early *Drosophila* embryo is a very convenient system for investigating the coupling between the spindle and the actomyosin cortex because of this organism's amenability to genetic analysis, inhibitor microinjection and microscopy [10]. In early *Drosophila* embryogenesis, some morphogenetic events such as cellularization [11] and nuclear migration [12] indicate interactions between the actomyosin cytoskeleton and microtubule arrays; myosin II is thought to have additional as yet unidentified functions [13]. Following our earlier efforts [14], here we focus on the syncytial blastoderm divisions that occur at the cortex of the embryo, just beneath the plasma membrane, where dramatic redistribution of the cortical actin accompanies spindle morphogenesis [15]. In these cycles, actin concentrates into ‘caps’ centered above each nucleus and centrosomes. As the nuclei progress into prophase, the centrosomes migrate toward opposite poles and the caps expand in synchrony with the centrosomes [14]. After NEB, a transient steady state is reached in prophase, after which the centrosomes separate and the spindle elongates further.

Here, we concentrate on the early stage of mitosis – prophase – because myosin-dependent contraction of the cortex has been reported at this stage, while at prometaphase myosin concentration starts to decline rapidly throughout the cortex [16]. The role of myosin II in cellularization [11] and the influence of astral MT arrays on the rapid spatial reorganizations of the actomyosin cortex [15], [17] are well documented. Actin dynamics must play an important role in centrosome separation based on the observations that separation is incomplete in *Drosophila* embryos treated with cytochalasin D [18] and that actin polymerization is crucial for the centrosome separation before NEB [19], but details of this cortex-to-spindle feedback and myosin II involvement were not studied.

**Figure 1:**
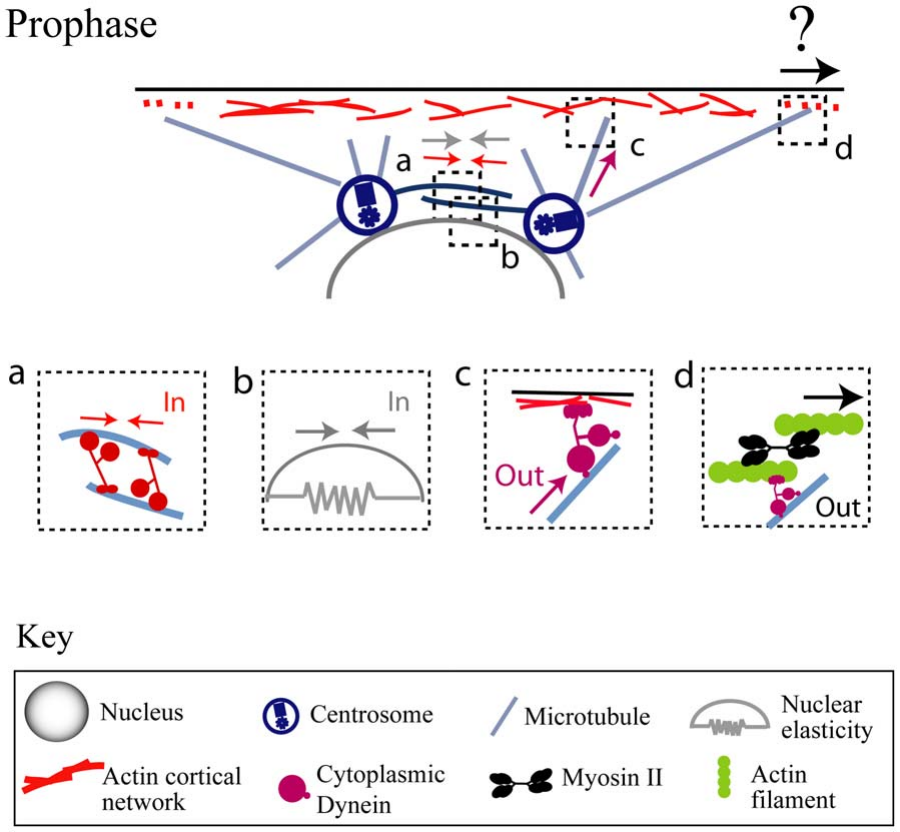
Force balance in prophase. Our previous work supports the hypothesis that in prophase the balance between the inward forces generated by kinesin-14 sliding interpolar MTs together (a) and nuclear elasticity (b) and the outward forces generated by dynein pulling on astral MTs (c) governs the spindle length. Here, we test the idea that an additional outward force is exerted by myosin-II (d) indirectly – by contracting the actin cortex and pulling outward on astral MTs cross-linked with the deforming actin network.

The question about the nature of the spindle-cortex interaction is intimately linked to another outstanding question – the relation between the internal and external forces shaping the spindle [20]. Recent work points to a principal role for a molecular motor-generated force balance in spindle assembly and pole-pole separation [14], [21], [22] (Fig. 1). Recent data and modeling suggest that cooperative interactions between multiple spindle associated internal mitotic motors must be complemented by external cortical dynein motors that pull out astral MTs to coordinate prophase spindle assembly. It is plausible that dynein anchored on the actin cortex can slide astral MTs against the cortex [23] and separate spindle poles. Based on time-lapse imaging, functional perturbation experiments and quantitative modeling, we hypothesized that the force-balance for spindle pole separation depends on outward forces generated by dynein associated with the actin caps being antagonized by inward forces exerted by the kinesin-14, Ncd and nuclear elasticity [14], [21] (Fig. 1).

Here we set out to further explore the molecular basis of this force-balance, asking whether myosin II contributes to the early spindle assembly, perhaps by generating a cortical flow that pulls actin radially outward, and with it the cross-linked astral MTs [2]. Time lapse microscopy of *Drosophila* embryos expressing fluorescently tagged myosin light chain, microinjected with rhodamine-tubulin was used to study and compare the dynamics and distribution of myosin II and microtubules. To examine the role of myosin II on spindle dynamics we used a myosin II inhibitor (the Rho kinase inhibitor, Y-27632 [16], [24]). In order to assess the role of F-actin in the early mitotic spindle, we used *Scrambled* (*Sced*) mutants which display defects in furrow formation and early centrosome separation [18], [25]. We observed, to our surprise, that myosin inhibition, rather than decreasing the spindle length, as expected from an outward myosin-dependent force, led to spindle lengthening. However, microscopy revealed that the actin caps expand when myosin II is inhibited, so dynein can exert a greater outward force in this case. We further report that in *Sced* mutants with disorganized actin caps, the pole-pole distance changes very little, and that it shortens significantly when myosin II is inhibited in these mutants. We propose that dynein and myosin II synergize in elongating the early mitotic spindle, albeit not directly. Rather, under circumstances when relatively rigid actin caps are present, myosin-II has little effect and dynein alone generates the outward force on spindle poles. However, if the actin cortex weakens, myosin contracts it locally and augments dynein in pulling out the astral MTs.

## Results

### Myosin inhibition in a *Drosophila* syncytial embryo results in greater centrosome separation during prophase

To test the hypothesis that myosin II plays a role in spindle dynamics, we microinjected a specific Rho kinase inhibitor, Y27632, into *Drosophila* syncytial embryos expressing GFP-tubulin. This drug disrupts myosin II activity by inhibiting Rho kinase which is responsible for myosin II activation via the serine and threonine phosphorylation [24]. Y27632 has been successfully used in previous studies to inhibit myosin function [16], [17] and its specificity for myosin II has been documented: depletion of DROK (Rho kinase in *Drosophila*) by either using RNAi or Y27632 gave identical results in S2 cells in preventing cell shape change, a process which depends on functional myosin [26]. In human cells, the use of Y27632 prevented myosin II localization at the cleavage furrow and generated defects in cytokinesis [27].

**Figure 2:**
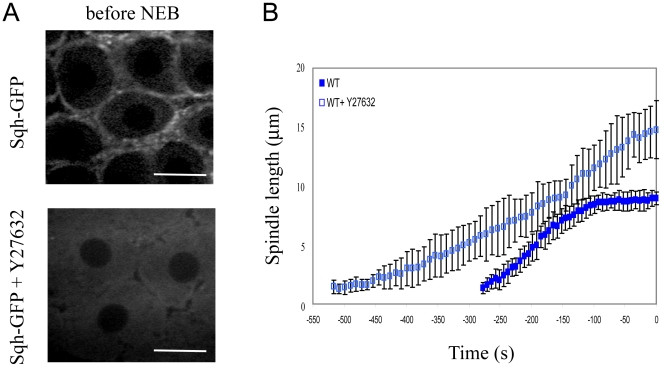
Effect of myosin inhibition on spindle development in WT embryos. A) Injection of the myosin inhibitor Y27632 causes myosin to lose its cortical localization. In Sqh-GFP expressing embryos the spaghetti-squash signal largely disappears from the cortex and becomes diffuse after Y27632 is injected. Bars, 10 µm. B) Graph of pole-pole distance as a function of time (NEB is t = 0 s) for GFP-Tubulin expressing embryos (WT) and myosin inhibited embryos (WT+Y27632). After myosin inhibition pole separation during prophase is greater than in WT.

Injection of Y27632 into Sqh-GFP expressing embryos disrupted myosin II localization, as shown by the disappearance of the myosin II regulatory light chain, spaghetti-squash, from the cortex (Fig. 2A), indicating that Y27632 is an appropriate inhibitor to study the function of myosin II during spindle morphogenesis in early embryogenesis. After microinjection of Y27632 in GFP-tubulin expressing embryos we found an increase in the distance between the two centrosomes during prophase compared to control embryo (Fig. 2B).

### Myosin II inhibition changes the actin distribution at the cortex

**Figure 3:**
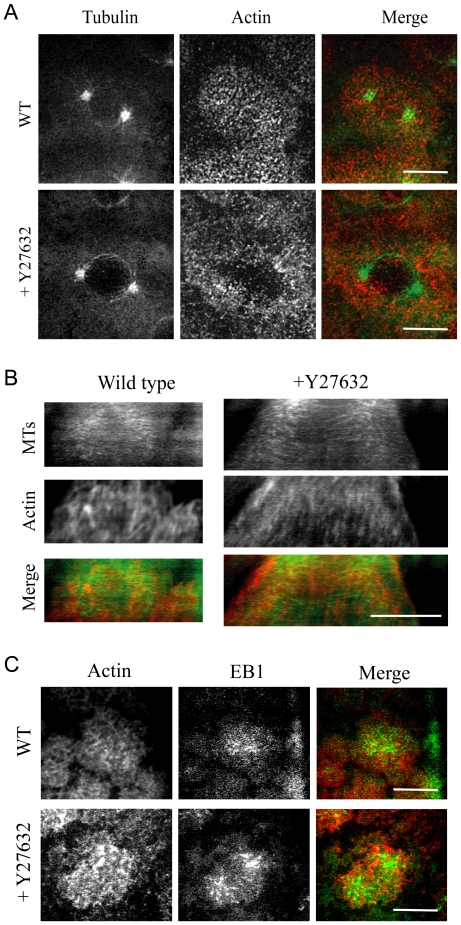
Effect of myosin inhibition on the dynamics of actin and MTs. A) Images from a time-lapse movie of a WT embryo expressing GFP-tubulin injected with rhodamine-actin and the myosin inhibitor Y27632. When myosin is inhibited, actin distribution in prophase changes compared to control showing a less compact and wider distributed actin structure above the nucleus (note the void area). Images are projections of 8–10 confocal planes taken 0.5 µm apart. B) Kymographs (from −250 to −100 seconds before NEB) of prophase embryos injected with rhodamine actin with or without Y27632 confirm that in the absence of myosin the outer edge of the actin cap expands at a faster rate and to a greater width than in control. Same confocal planes taken at the very top of the actin cap were imaged for both actin and MTs. Red, actin; green, tubulin. C) Images from a time-lapse movie of GFP-EB1 expressing embryos injected with rhodamine-actin with and without Y27632 during prophase. Tracking of MT tips shows that both in WT and myosin-inhibited embryos, MT tips uniformly reach the surfaces of both narrower and wider actin caps (EB1-GFP, green; rhodamine-actin, red). Bars, 10 µm.

To see whether myosin perturbation affected actin structure and distribution, we co-injected rhodamine-actin with the myosin inhibitor. In addition to the increased pole-pole separation described before, we observed a dramatic change in F-actin distribution and organization before NEB (Fig. 3A). In control embryos, F-actin is organized into a compact actin cap right above the nucleus/centrosomes in prophase. The most striking effect of the myosin inhibition is the increment of the actin cap area suggesting that myosin II is playing a role in restricting the actin expansion (see Discussion).

To gain more insight into the actin expansion process at the cortex in both control and myosin-inhibited embryos, we analyzed kymographs of cortical actin along the pole-pole axis (Fig. 3B). We saw that the actin cap expands faster and to a greater extent in myosin-inhibited embryos, and observed ‘streaks of actin’ in the kymographs expanding outward with respect to the position of the centrosomes in both WT and myosin-inhibited embryos. These streaks had a much higher slope in myosin-inhibited embryos indicating a faster expansion of the cortical actin array. Note that we cannot distinguish at this point between physical centrifugal F-actin movements and outward polymerization waves near the cap edge. Indeed, in some cases it was also possible to see nascent spots of F-actin polymerized along the cap edge in the direction of the actin expansion, perhaps suggesting that MTs reaching that area promote actin polymerization which is responsible for the growth of the actin cap.

**Figure 4:**
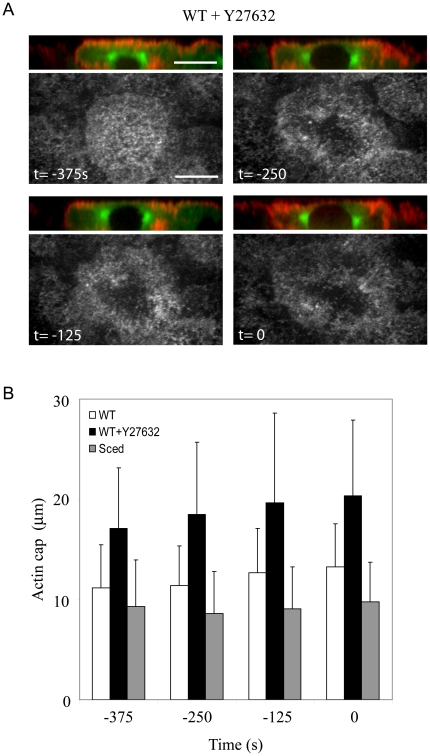
Quantification of the actin cap size. A) Side views of actin, nucleus and centrosomes (rhodamine-actin, red; GFP-Tubulin, green) and corresponding top views of actin in prophase WT embryos in the presence of the myosin inhibitor Y27632 reveal that the centrosomes segregate around the nucleus, likely being pulled outward by the increasing forces. The resulting vertical component of the force pushes the nucleus upward against the actin cap thinning the cortex and creating the ‘hole’. NEB is t = 0 s. Bars, 10 µm. B) The square root of the actin cap areas as functions of time (NEB is t = 0 s) in WT embryos in the absence and presence of the myosin inhibitor Y27632 and in the *Sced* embryos.

We also noticed the formation of a “hole” in the center of the actin cap in myosin-inhibited embryos (Fig. 3A). Its origin could be the steric hindrance of the nucleus being pushed up into the cortex with a greater force coming from a stronger outward pull on the centrosomes (Fig. 4A, also see below). We also measured the average thickness of the actin cap right above the nuclear top in control and myosin-inhibited embryos and found that the thickness decreased from 2.2±0.3 µm in control to 1.25±0.4 µm in myosin-inhibited embryos. To quantify the expansion of the actin cap in the myosin-inhibited embryos, we measured the areas of the actin caps as functions of time in control and myosin-inhibited embryos. The square root of the areas characterizes the cap sizes: for almost circular caps the square root of the area is close to the cap diameter. Both cap size and spindle length increase with myosin inhibition (Fig. 2B, 4B). The cap size also increases with time in myosin inhibited embryos in sync with spindle length growth (Fig. 4B).

### Astral MTs reach the increased actin cap area in myosin-inhibited embryos

Dynein co-localizes with the actin cortex [14], [21], [28], and its inhibition decreases spindle elongation during prophase and prometaphase [21]. Modeling of the available data from perturbation experiments strongly suggests that during prophase dynein is the outward force generator [14], [22]. Thus, it is possible that the cause of the increment in pole-pole distance after myosin inhibition is due to astral MTs reaching the increased actin cap area and interacting with a greater number of dynein molecules, resulting in a stronger outward force on the spindle poles. To see if this is plausible, we examined the distribution of actin and MTs at the cortex (Fig. 3C) by checking whether dynamic MT plus ends were able to reach the whole cortex after myosin II inhibition.

To do this, we followed MT plus end localization with respect to actin at the cortex by injecting rhodamine-actin into embryos expressing the plus-end tip-tracker protein, EB1-GFP (Fig. 3C). In WT embryos, we could see spots of EB1-GFP transiently contacting the cortex almost evenly wherever actin was present. Even when myosin was inhibited, these EB1-GFP spots were able to reach uniformly the wider distributed cortical actin and showed a distribution similar to WT embryo, over a greater area of the expanded actin cap. This observation suggests that myosin II inhibition does not affect the ability of MT plus ends to reach the cortex and possibly generate pulling forces. In addition, no significant difference in the density of the EB1 spots were observed between wild type and myosin inhibited caps (# tips/actin area: WT, 159.6±32.3; WT+Y27632, 136.9±54.9). We hypothesize that myosin inhibition does not change the density of the dynein force generators (number of motors pulling on the astral MTs per unit area of the cap). Thus, the total outward pulling force is greater in the myosin-inhibited embryos because the cap area is greater.

### A change in actin distribution is not sufficient to impair early spindle development but shows an additive effect in combination with myosin inhibition

To test further whether the cortical area had any effect on the force balance required for centrosome separation, we used a mutant for *Sced* protein, which shows defective actin organization at the cortex: in *Sced* mutants, smaller actin caps and absence of actin furrows were reported [18], [25]. Although the *Sced* protein has not been shown to be closely related to sequences currently in the non-redundant or expressed sequence tag (EST) databases, it has been proposed, based on its localization to centrosomes and mitotic furrows, that *Sced* could be involved in actin reorganization via a MTs-independent process either by direct recruitment of actin or by promoting the localization of actin polymerization factors [18].

**Figure 5:**
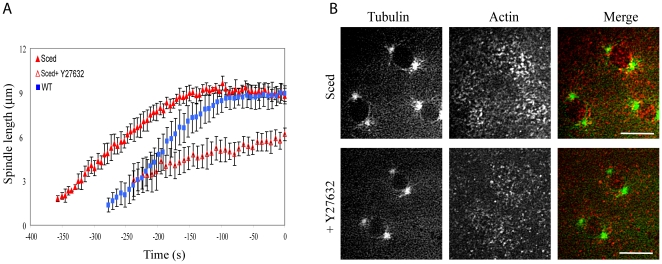
Effect of myosin inhibition on spindle development in Sced embryos. A) Pole-pole distances in prophase as functions of time (NEB is t = 0 s) show a different degree of pole separation in *Sced* embryos in the presence and absence of the myosin inhibitor Y27632. The pole-pole separation in *Sced* embryos is comparable to that in control, but when myosin is inhibited, centrosomes separate less than in control and *Sced* embryos. B) Images from a time-lapse movie of a *Sced* embryo in prophase co-injected with FITC-tubulin and rhodamine-actin in the presence or absence of Y27632. *Sced* embryos have smaller and less organized actin caps which become even less compact when myosin is inhibited. Images are projections of 8–10 confocal planes taken 0.5 microns apart. Red, actin; green, tubulin. Bars, 10 µm.

The *Sced* mutant, which shows a strong phenotype in the early syncytial divisions in *Drosophila* [18], allowed us to test the effect of a reduced actin cap area, from where dynein could pull on MTs, without the use of any drug. *Sced* mutants have smaller actin caps (Fig. 4B). Therefore, we expected to see a decrease in pole-pole separation as a consequence of a decrease in forces acting on the poles from the cap. We followed the spindle pole dynamics in *Sced* embryos before NEB, and we did not find a significant difference in the pole-pole distance between WT and *Sced* mutant embryos in late prophase (Fig. 5A). The average cap thickness above the nucleus also changed very little (2.2±0.3 µm in control versus 2.0±0.3 µm in *Sced* embryos). Note though that the centrosomes start to separate earlier in mutants. However, when we inhibited myosin II in *Sced* mutants, the pole-pole distance was reduced compared to both WT and Sced embryos (Fig. 5A). Actin structures were also affected in *Sced* mutants in the absence of myosin: the actin cap became smaller and less defined (Fig. 5B).

**Figure 6:**
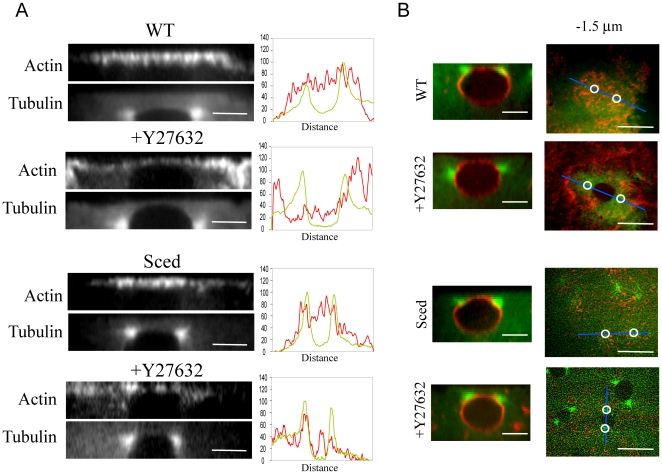
Effect of myosin inhibition on actin distribution in WT and Sced embryos. A) Side views of actin at the cortex in WT and *Sced* embryos in the absence and presence of the myosin inhibitor Y27632 reveal different actin distributions and cap sizes. The WT actin cap is compact with an evenly distributed thick layer of actin. When myosin is inhibited, the cap expands remaining thick at the margins and becoming thinner in the center. Note that the nucleus ascends ‘indenting’ the cap. In *Sced* embryos the cap becomes smaller compared to WT embryos, and when myosin function is inhibited, the actin in the cap becomes disorganized. Corresponding line scans of fluorescence intensity (tubulin, green; actin, red) taken along the actin cap in relation to the centrosomes' position (taken along the pole-pole axis), confirm the qualitative description of the changes in actin distribution. Bars, 5 µm. B) Side view of the centrosomes' position with respect to the nucleus. The nuclear membrane was stained in vivo with fluorescent conjugated WGA in WT embryos expressing GFP-tubulin or in *Sced* embryos injected with FITC-tubulin. When myosin is inhibited in WT, the centrosomes move further down and around the nucleus than in control. In *Sced*, the centrosomes are positioned slightly more apically, and when myosin in inhibited, this apical shift and lesser separation becomes more evident. Bars, 10 µm. Next to the side views are corresponding images of a wild-type embryo expressing GFP-tubulin (green) injected with rhodamine-actin (red) and a *Sced* embryo injected with FITC-tub (green) and rhodamine-actin (red) in the presence and absence of the myosin inhibitor Y27632. The prophase actin cap taken 1.5 µm below the top shows the difference in actin density and distribution between wild-type and *Sced* embryos in the presence or absence of myosin. Circles mark the position of the centrosomes which in some cases are not clearly visible. Bars, 10 µm.

To probe deeper into the apparent puzzle as to why the spindle length remained the same in *Sced* embryos, where actin is disorganized, we undertook a detailed analysis of actin organization in WT, *Sced* mutant and myosin-inhibited embryos by looking at the distribution of actin in the cap with respect to the centrosomes. Fig. 6A shows side views of the actin cap during prophase in WT and *Sced* mutants with and without myosin inhibition. In *Sced* mutant embryos actin covers a smaller area than in control. In WT myosin-inhibited embryos, actin accumulates at the periphery and expands forming small furrows. In *Sced* embryos in the absence of myosin action, the cap expands outward while the actin density fluctuates in space becoming patchy (small, micron-size patches of lower density are intermittent with patches of higher density). Combined line scans of actin signal taken along the cortical actin layer and tubulin intensity taken across the two centrosomes quantitatively confirm these qualitative differences in actin cap distributions.

Finally, we examined the position of the centrosomes relative to the nucleus in prophase and found that in WT the centrosomes separate further around the nucleus in the absence of myosin function, while the nucleus itself appears to be pulled up indenting the actin cap (Fig. 6B). This could be an indication of a greater pulling outward force applied by the astral MTs on the centrosomes [14]. Sometimes, detached centrosomes could also be seen in myosin-inhibited WT embryos (data not shown and [19]) lending further support to the hypothesis of a stronger pulling force. Note that the top view sometimes shows a larger nucleus in a myosin-inhibited embryo, however, the side view clearly indicates that the centrosomes have moved further down along the nuclear surface, so that the nuclear cross-section in the centrosomal plane is greater. In fact, the nuclear volume does not seem to change in the myosin-inhibited embryos. This observation is in contrast with the result reported in [19], where the increased inter-centrosomal distance in myosin-inhibited embryos was observed and interpreted as nuclear swelling.

## Discussion

Earlier studies suggested that the myosin-powered contraction of the cortex can generate a force on the centrosomes by pulling on the astral MTs cross-linked with the actin cortex [2]. For example, MTs were observed to undergo directed movement along cortical actin filaments during wound healing experiments in Xenopus oocytes [29] and along polarized actin cables in yeast [30] (in the latter case, the Myosin-II-Kar9-Bim1 complex is responsible for this transport). However, the nature of specific actin-myosin-MT interactions in various systems and organisms remains an open question.

**Figure 7:**
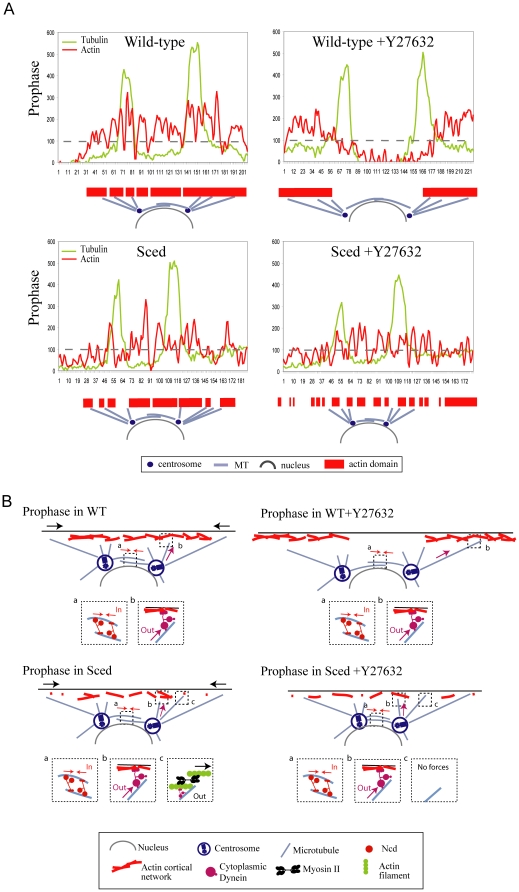
Synergistic actions of actin, myosin and dynein in spindle development. Hypothesis for each condition (B) is based on the actin distribution as shown in the corresponding line scans (A). The intensity value of 100 has been chosen as the arbitrary cut-off value to discriminate between the ‘soft’ (below 100) and ‘firm’ (above 100) actin patches. In the WT embryo, most of the actin cap is firm, and dynein pulls from the large firm actin cortex (inset b) while kinesin-14 exerts an inward force (inset a). When myosin is inhibited, the area of the firm actin is shifted outward focusing the dynein pulling in the outward direction. In the *Sced* embryo, many soft actin patches appear, but myosin contracts actin filaments there (inset c) complementing the pulling action of dynein (inset b). When myosin is inhibited in the *Sced* embryo, dynein pulls from the small infrequent firm patches of actin and does not separate the poles effectively.

Our observations support the hypothesis that in the early *Drosophila* embryo during prophase, dense actin patches provide a scaffold from which dynein can pull astral MTs outward promoting pole-pole separation, while myosin regulates the actin cap size (Fig. 7, WT and WT+Y27632). However, when the actin distribution is compromised, myosin can exert local mechanical action on the loose weakened actin patches and generate contraction that pulls astral MTs cross-linked with actin filaments (Fig. 7, *Sced*). This myosin pulling from the loose actin patches synergizes with the dynein pulling from the remaining dense actin patches, so that the pole-pole separation in *Sced* embryos is almost normal. However, when both myosin and actin are compromised, more ‘weakened actin areas’ are formed at the cortex, thus, the dynein-generated pulling force from the remaining firm actin patches (Fig. 7, *Sced*+Y27632) is decreased resulting in an incomplete pole-pole separation.

This hypothesis suggests that myosin II participates in regulating the actin cap geometry and dynamics in two ways. First, myosin antagonizes the spreading of the cap outward, but does not disrupt the actin assembly within the cap. This effect is unlikely to be based on centripetally contracting the cap because myosin and actin in the early WT embryo are largely separated in space [11], [15] and no significant deformations of the actin caps are observed in WT embryos. More likely, myosin facilitates disassembly of actin filaments [31] at the rim of the cap or acts by a combination of this and other recently uncovered mechanisms in different systems [32], [33].

Earlier we reported that dynein co-localizes with the actin cortex and that dynein inhibition leads to a severe spindle shortening in prophase [14], [21]. We propose that when the actin assembly is normal within the cap, then dynein alone is responsible for the generation of the outward force in WT embryos: dynein motors pull on astral MTs effectively from the dense and robust undeformed cap. When myosin II is inhibited, the dense actin cap area expands outward, which probably focuses the dynein-mediated pulling in the outward direction increasing the outward force and spindle length, as observed.

We hypothesize that when the actin dynamics in the cap is disrupted, the F-actin assembles throughout the cap with an uneven density, so that high density (and likely mechanically firm) patches of actin network are interspersed with low density (likely mechanically soft) patches. We call such a cap ‘loose’. We propose that dynein can effectively pull from the dense actin patches, but not from the soft ones, because the result of such pulling would be local deformations of the cortex, rather than ‘reeling in’ the astral MTs (Fig. 7B). However, myosin II motors, which cannot deform the dense actin patches, are able to locally deform the soft patches [34], and astral MT tips reaching and associating with the F-actin in these soft patches can be pulled outward by these myosin-caused upward deformations (Fig. 7B). Therefore, on the loose actin cortex dynein and myosin synergize in the generation of outward force. This model predicts that the outward force and thus the spindle length remains unchanged if the actin cap is loose, because diminished dynein action is supplemented by the local myosin contraction. If myosin is inhibited in this situation, then the force becomes weaker and the spindle – shorter. To test this idea, we investigated *Sced* mutants that have a loosely organized actin layer at the cortex (Fig. 7A) with functional myosin II, and discovered that the spindle pole-pole distance in these cortical mutants was similar to that in WT embryos. Myosin inhibition in these mutants indeed led to spindle shortening.

The model we are proposing (Fig. 7B) does not address the very complex question about the mechanical and biochemical regulatory interactions of astral MTs with the actin-myosin cortex in the early *Drosophila* embryo. There are too many possible interactions [1] and not enough data to discriminate between various possibilities. For example, MTs can transport either F-actin itself or actin-nucleating agents to the cap [17], as well as molecules regulating myosin contraction [1] and myosin itself [35]. Myosin II could be concentrated at the cap's margins because both actin and myosin are transported along astral MTs growing along the cortex towards their plus ends, with actin and myosin exhibiting different affinities for the cell's cortex [15]. There is also the possibility of a feedback loop from actin-myosin to MTs [36]. The nature of these MT-actin-myosin interactions will have to be addressed in future studies. Recently, it was observed [37] that the membrane and actin-myosin cortex deformed significantly by MT-mediated pulling forces when the cortex was weakened in one-cell C. elegans embryos, consistent with the hypothesis that the MTs need to be anchored to a stiff actomyosin network to be able to transduce forces. We did not detect clearly such large scale deformations, but further, higher resolution, microscopy in *Drosophila* embryos will be needed to investigate a possibility of a similar phenomenon. We did, however, noticed patches of actin at the side of the nucleus below and near the centrosomes in the myosin- inhibited embryo (see side views in Fig. 4A). These patches could indicate large-scale cortex deformations when greater pulling forces are applied to the cortex.

To conclude, we suggest that there is a complex synergy between dynein and myosin II actions before NEB (Fig. 7B): dynein pulling on astral MTs from the dense and firm parts of the actin cortex is responsible for most of the outward force elongating the spindles, while myosin II regulates the cap size and supplements the dynein-generated force when actin organization is compromised locally by contracting the weakened cortical patches and pulling on the actin-cross-linked astral MTs. Further analysis is required to understand the precise molecular mechanism by which myosin-II contributes to other stages of spindle development in the *Drosophila* embryo and other organisms.

## Materials and Methods

### Fly Stocks

Flies were maintained and 0 to 2 h embryos were collected as described [38]). Experiments were performed using embryos expressing GFP-Tubulin (provided by Dr. Allan Spradling, Carnegie Institute, Washington), EB1-GFP (provided by Dr Steve Rogers, University of North Carolina at Chapel Hill, Chapel Hill, NC), GFP-tagged myosin II light chain and *Sced* embryos (provided by Dr. Wiliam Sullivan, UCSC, Santa Cruz, California).

### *Drosophila* embryo injection

Microinjection of 0–2 h embryos was performed as described [39]. Briefly, as indicated for each experiment, rhodamine conjugated monomeric actin (Cytoskeleton, Denver, CO) was injected into GFP-tubulin expressing embryos but in the case of Sced, FITC-tubulin and rhodamine-actin were coinjected. The myosin inhibitor, Y27632 was used at the concentration of 50 mM in the needle.

### Image acquisition

Time lapse confocal images were acquired on an Olympus (Melville, NY) microscope equipped with an Ultra View spinning disk confocal head (Perkin Elmer-Cetus Wallac, Inc., Gaithersburg, MD) and a 100× 1.35NA objective. Images were analyzed using MetaMorph Imaging software (Molecular Devices, Downintown, PA). Quantitation of spindle pole positions was carried out using MetaMorph. The positions of the two poles were logged, and the distance between them was calculated and plotted as a function of time using Excel.
